# Trials evaluating nicorandil renoprotection against contrast-induced nephropathy after coronary angiography or percutaneous coronary intervention: a systematic review and meta-analysis

**DOI:** 10.1186/s43044-025-00705-4

**Published:** 2025-11-16

**Authors:** Nestor Lemos Ferreira, Débora Regina Aguiar, Sachin G. Nair, Mohamed Ashraf Shehab, Abiodun Bamidele Adelowo, Mohammad Rafi Damirchi, Dan Jones, Krishna Rathod, Zahid Khan

**Affiliations:** 1 Hospital Sirio Libanes, Sao Paulo, Brazil; 2 Universidade Federal de Sao Paulo. Escola Paulista de Medicina, Sao Paulo, Brazil; 3 Amrita Institute of Medical Sciences and Research Center, Kerela, India; 4 Menoufia University, Menoufia, Egypt; 5 Niger Delta Power Holding Company, Asokoro, Abuja, Nigeria; 6 Golestan University of Medical Sciences, Gorgan, Iran, Islamic Republic of; 7 Bart’s Heart Centre, London, UK; 8 Queen Mary University, London, UK

## Abstract

**Background:**

Ischaemic heart disease (IHD) is a leading cause of mortality and morbidity globally. Coronary angioplasty has a vital role in treating coronary artery disease. However, this is associated with a small risk of serious side effects, including contrast-induced nephropathy, vascular complications and arrhythmia. Contrast-induced nephropathy (CIN) is a serious and common complication of coronary angioplasty that can lead to renal failure and major adverse cardiac and renal outcomes.

**Methods:**

We conducted a systematic review and meta-analysis by searching multiple databases, including PubMed, Scopus, Embase, Google Scholar, and ScienceDirect, as well as other sources. The inclusion and exclusion criteria are described in detail later in this article. Two independent reviewers performed the literature search in September 2024 and identified 282 articles. The study was conducted following the population, intervention, comparator, and outcome (PICO) framework and the Preferred Reporting Items for Systematic Reviews and Meta-Analyses (PRISMA) guidelines. A total of 17 studies were included in the final analysis after applying the inclusion and exclusion criteria. The exclusion criteria were guidelines, case reports, qualitative research, and letters to the editor, commentaries, conference proceedings, gray literature, opinions, policy papers, and case series. Articles published after 2010 were included in this meta-analysis, and data analysis was performed using Rayyan statistical software.

**Results:**

This study demonstrated that nicorandil was associated with protective effects against CIN. The total number of patients in the Nicorandil and placebo groups were 3836 and 3858 respectively. The occurrence of CIN was 5.14% in the nicorandil group, compared with 13.15% in the control group. This study also confirmed the dose-dependent effect of nicorandil on CIN. Among 662 patients enrolled in three studies, 3,9% in the double dose (DD) group presented with CIN, compared with 8,4% in the standard dose (SD) group. The occurrence of MACE was 5.7% in the Nicorandil group and 8.2% in the control group. However, there was no statistically significant protective effect against major adverse cardiovascular events (MACE) or major adverse kidney events (MAKE). Only a few studies measured the impact on MAKE, and the findings may not be truly representative of its effects.

**Conclusion:**

This study demonstrated the renoprotective effects of nicorandil in preventing CIN in patients undergoing coronary angioplasty, and this relationship was also evident from the double-dose response. Further larger size randomised controlled trials are recommended to assess the efficacy of nicorandil in preventing CIN in patients undergoing coronary angioplasty.

## Introduction

Ischaemic heart disease (IHD), also known as coronary artery disease (CAD), is the most prevalent cardiovascular disease and the leading cause of disability and mortality globally 9 [1–3]. It accounted for more than 180 million Disability-Adjusted Life Years (DALYs) in 2019 [4], approximately 720,000 cases, and over 9 million deaths globally every year [5]. Despite advances in medical care, the burden of IHD is increasing globally, thus making it a significant threat to global public health and attracting a large percentage of annual global healthcare expenditure [4, 5].

Prompt treatment of IHD with coronary angioplasty reduce the risk of patient’s mortality and comorbidity resulting in improved quality of life [6, 7]. Nevertheless, coronary angioplasty also has complications and some of these complications are related to the contrast media such as iodine and gadolinium used during coronary angioplasty. These media are also used in diagnostic tests such as computed tomography myocardial perfusion imaging (CT-MPI), coronary CT angiography (CCTA), coronary angiography (CAG), or percutaneous coronary intervention (PCI) [5, 8–11].

Contrast media induced nephropathy, allergic reaction, anaphylaxis, renal replacement therapy, major adverse cardiovascular and renal outcomes are some of the serious side effects observed in patients [8, 9]. Certain patients’ characteristics such as anemia, chronic kidney disease, diabetes mellitus, dehydration, volume of contrast used, advanced age, heart failure, multiple myeloma, hypoalbuminemia, liver cirrhosis, concomitant use of nephrotoxic drugs is more likely to predispose patients to CIN [5, 12, 13].

CIN can be defined as an increase in serum creatinine by ≥ 0.5 mg/dL (44 µmol/L) or by >25% from baseline within 48 h after the administration of radiocontrast media (RCM), without any other attributable causes [12, 14, 15]. CIN is the third most common cause of hospital-acquired ARI, responsible for approximately 12% of ARI cases, although the incidence may be as high as 24%, depending on the risk factors [15]. Various proposed mechanisms responsible for CIN included enhanced cellular damager due to reactive oxygen species, reduced intramedullary renal blood flow due to renal vasoconstriction caused by imbalance of local vasoactive mediators such as adenosine, nitrous oxide, endothelin, prostaglandin and reactive oxygen species that are released by the vascular endothelium in response to the contrast media induced cytotoxicity. The vasoconstriction effect seems to be more common in patients with chronic kidney disease [14–16].

Some of the recommended measures to reduce the risk of CIN include pre-hydration mainly in patients with chronic kidney disease (CKD), low-osmolar iodinated contrast media (ICM), and the use of prophylactic statins, low volume of contrast, stopping nephrotoxic drugs such as metformin before coronary angioplasty and reassessing renal function tests in one week post procedure [14]. Certain agents such as Nicorandil, nitrates and nitrates, ascorbic acid, theophylline, N-acetyl cysteine, high dose statins, and Prostaglandin E1 have been used to minimize the risk of CIN [17–20]. Nicorandil is an anti-anginal medicine owing to its ability to dilate blood vessels through potassium channel activation and its nitrate moiety [21]. This meta-analysis aimed at determining whether nicorandil can prevent CIN.

## Objectives

The primary endpoint was to evaluate whether nicorandil exerted nephroprotection against CIN in patients undergoing CAG or PCI. The secondary endpoint was to identify major adverse events (MAE) and major adverse kidney events (MAKE) associated with CAG or PCI.

## Methodology and material

This systematic review and meta-analysis were conducted using the 2020 Preferred Reporting Items for Systematic Reviews and Meta-Analyses (PRISMA) guidelines and the PICO framework (Table 1). The study is registered with PROSPERO under registration number CRD42024567009.

**Table 1:** Population, intervention, comparison, and outcome (PICO) framework

Variables	Definition
Population	Patients undergoing CAG or PCI
Intervention	Administration of Nicorandil
Comparison/Control	Control group on placebo treatment
Outcome	Reduction of CIN events and evidence of renal protection

### Data source and search strategy

**Fig. 1:**
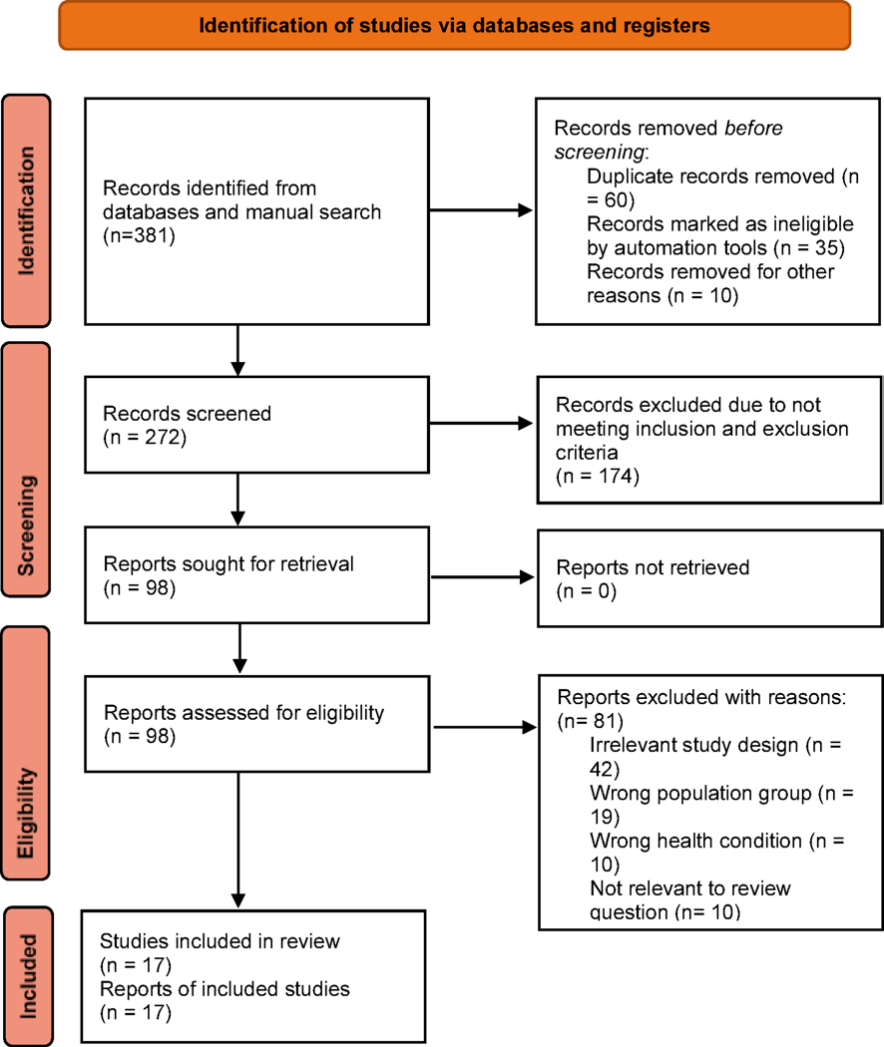
Preferred Reporting Items for Systematic Reviews and Meta-Analyses (PRISMA) flow diagram showing included studies in this meta-analysis

The search was performed across the PubMed, Scopus, Embase, Google Scholar, and ScienceDirect databases. Some of the searched items included Nicorandil, Contrast-Induced Nephropathy, Acute Renal Injury, chronic kidney disease, impaired renal function, renal insufficiency, renoprotection, ischaemic heart disease, cardiac catheterisation, computed tomography, myocardial perfusion imaging (CT-MPI), coronary CT angiography (CCTA), coronary angiography (CAG), and percutaneous coronary intervention (PCI). The search was performed in September 2024 by two independent reviewers, identifying two hundred eighty-two (282) related articles. The articles were reduced to 49 after duplicate analyses were performed using the Rayyan App. After applying the inclusion and exclusion criteria, 17 articles were included in the study, as shown in the PRISMA chart (Fig. 1). All included articles were published between 2011 and 2023. All discrepancies were resolved through consensus.

### Inclusion criteria

The inclusion criteria were as follows: randomised controlled trials (RCTs), observational studies, case-control studies, cohort studies, systematic reviews, and meta-analyses; full articles; articles written in the English language; and articles on adult human populations regarding the effect of nicorandil, compared to placebo, on preventing CIN nephropathy after CAG/PCI.

### Exclusion criteria

The exclusion criteria were as follows: guidelines, case reports, qualitative research, and letters to the editor, commentaries, conference proceedings, gray literature, opinions, policy papers, and case series.

### Data collection process and data items

The literature search was performed by two reviewers and extracted data was recorded in a standardised data abstraction form (Microsoft Excel^®^). Duplicates were removed using the Rayyan software. The data collected included title, authors, year of publication, study population, intervention group, control group, follow-up period after the procedure, results in terms of CIN events comparing nicorandil and placebo, as well as nicorandil double dose (DD) and nicorandil standard dose (SD), and the analysis of nicorandil regarding MAE, which were considered as follows: nonfatal MI, revascularization, stroke, coronary artery bypass graft surgery, congestive heart failure, worsening of heart failure, pulmonary oedema, ventricular fibrillation, cardiac death, and gastrointestinal bleeding. End-stage kidney disease and temporary renal replacement therapy were the circumstances encompassed by MAKE.

### Statistical analysis

**Fig. 2:**
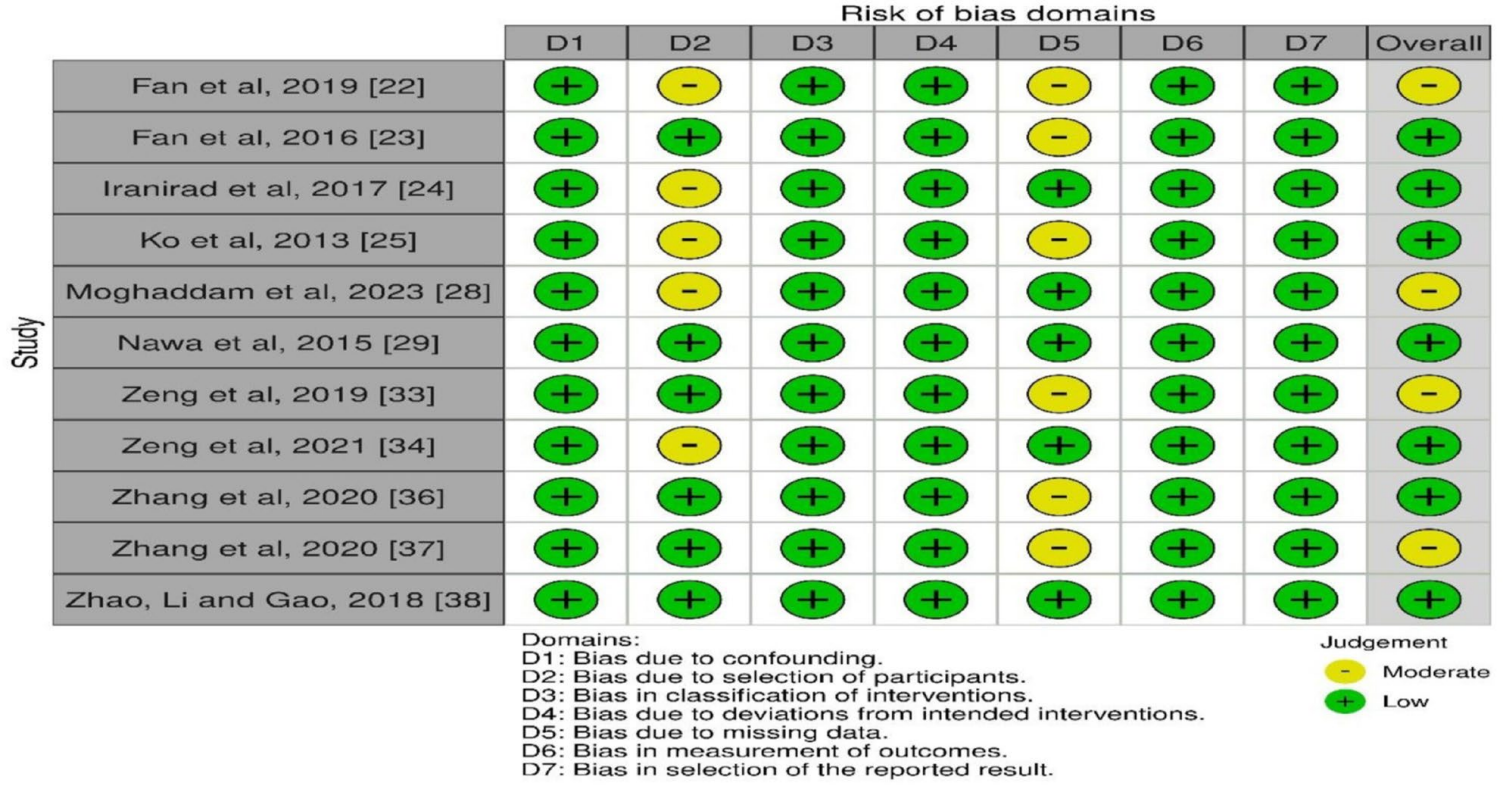
Risk of bias assessment for the studies included in this systematic review and meta-analysis

**Fig. 3:**
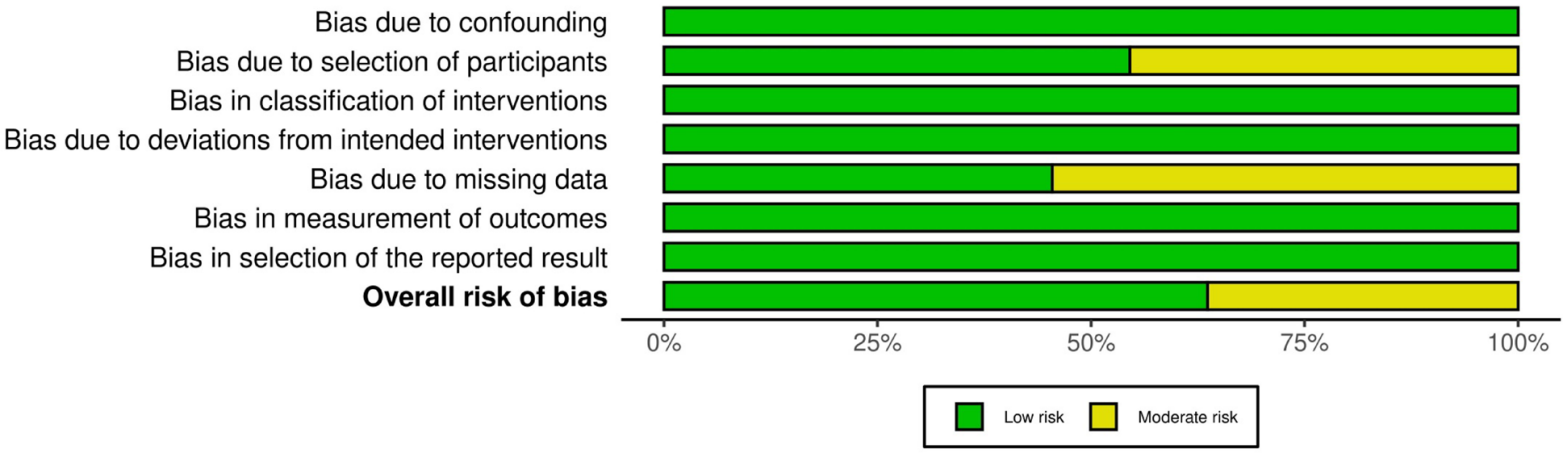
Risk of bias assessment plot

Due to low heterogeneity among studies, statistical analysis was performed using the Cochrane Review Manager (RevMan) 5.3 (The Cochrane Collaboration, Copenhagen) with dichotomous data and the fixed-effect model. Having said that, this analysis mitigated the risk of assigning greater weight to the results of small-sample research. The Risk of Bias evaluation was conducted using the Cochrane Risk of Bias 2 (RoB) tool (Figs. 2 and 3), and publication bias was assessed using funnel plots. The AMSTAR-2 tool was used to determine the quality of the systematic reviews and meta-analyses in this study, categorising them as low, moderate, or high quality (Table 2).

**Table 2:** A measurement tool to assess systematic reviews 2 (AMSTAR 2) tool for assessing the risk of bias in systematic reviews and meta-analysis

Study	Score	Date of analysis
Li et al., [26]	High quality	12/25/2024
Ma et al., [27]	Low quality	12/25/2024
Pranata et al., [30]	Low quality	12/25/2024
Sharp et al., [31]	Moderate quality	12/25/2024
Wang et al., [32]	Low quality	12/25/2024
Zhan et al., [35]	High quality	12/25/2024

Table 2 presents the AMSTAR-2 tool for assessing risk of bias in systematic reviews and meta-analyses.

## Results

**Fig. 4:**
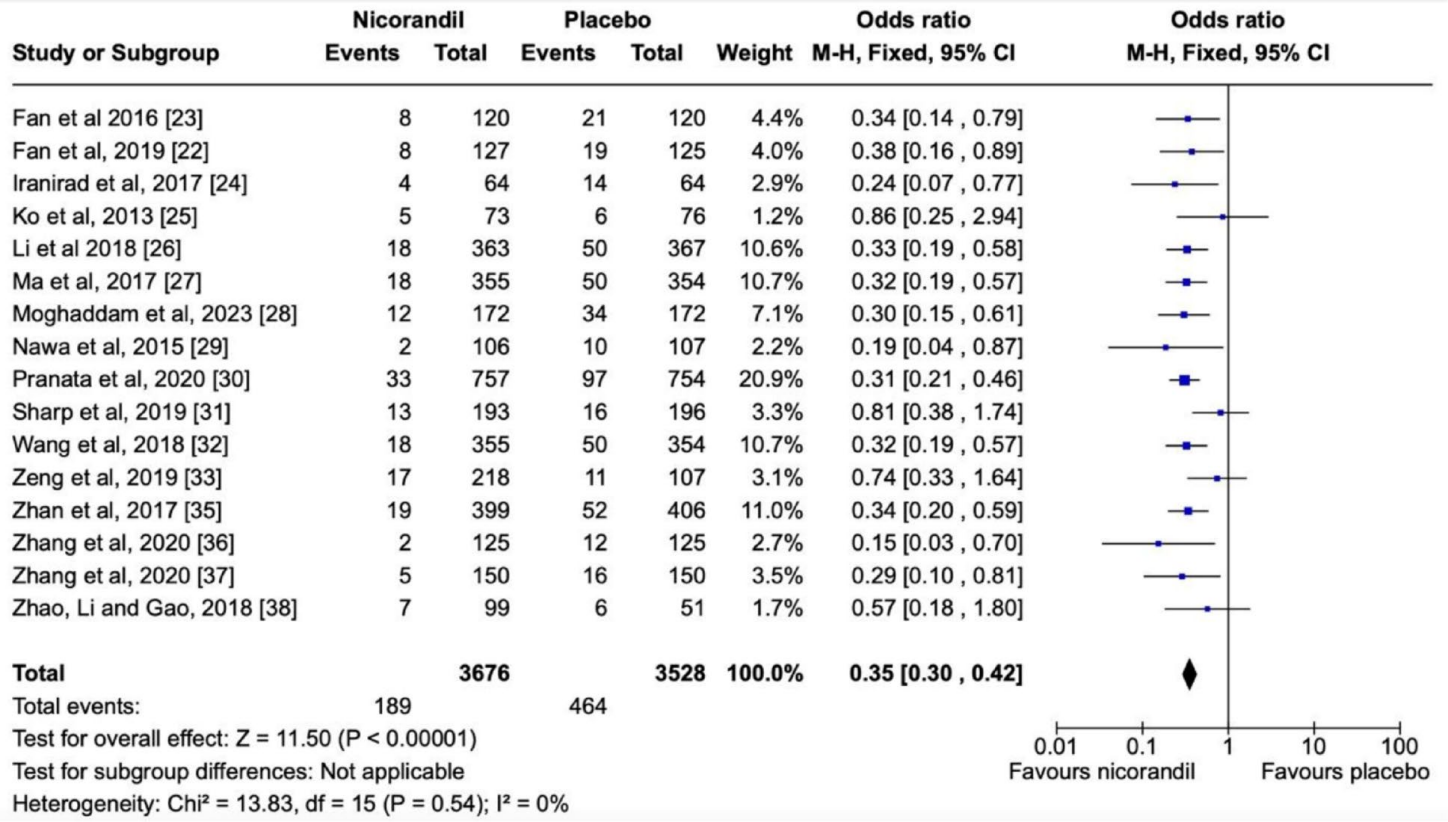
Forest plot showing the protective effect of nicorandil versus placebo against CIN

**Fig. 5:**
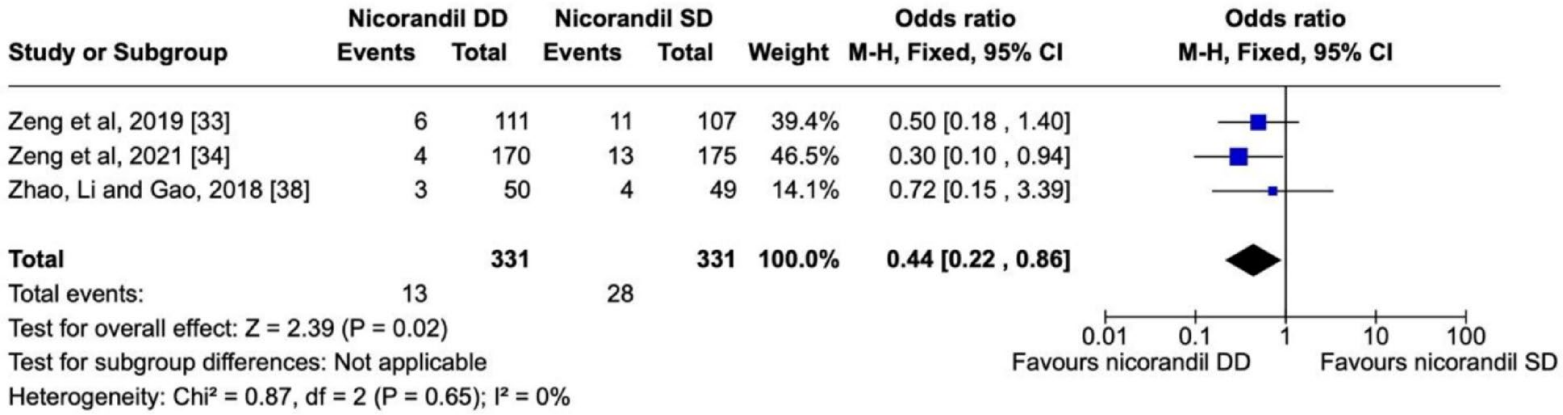
Forest plot showing positive dose dependent effect of nicorandil versus placebo against CIN

**Fig. 6:**
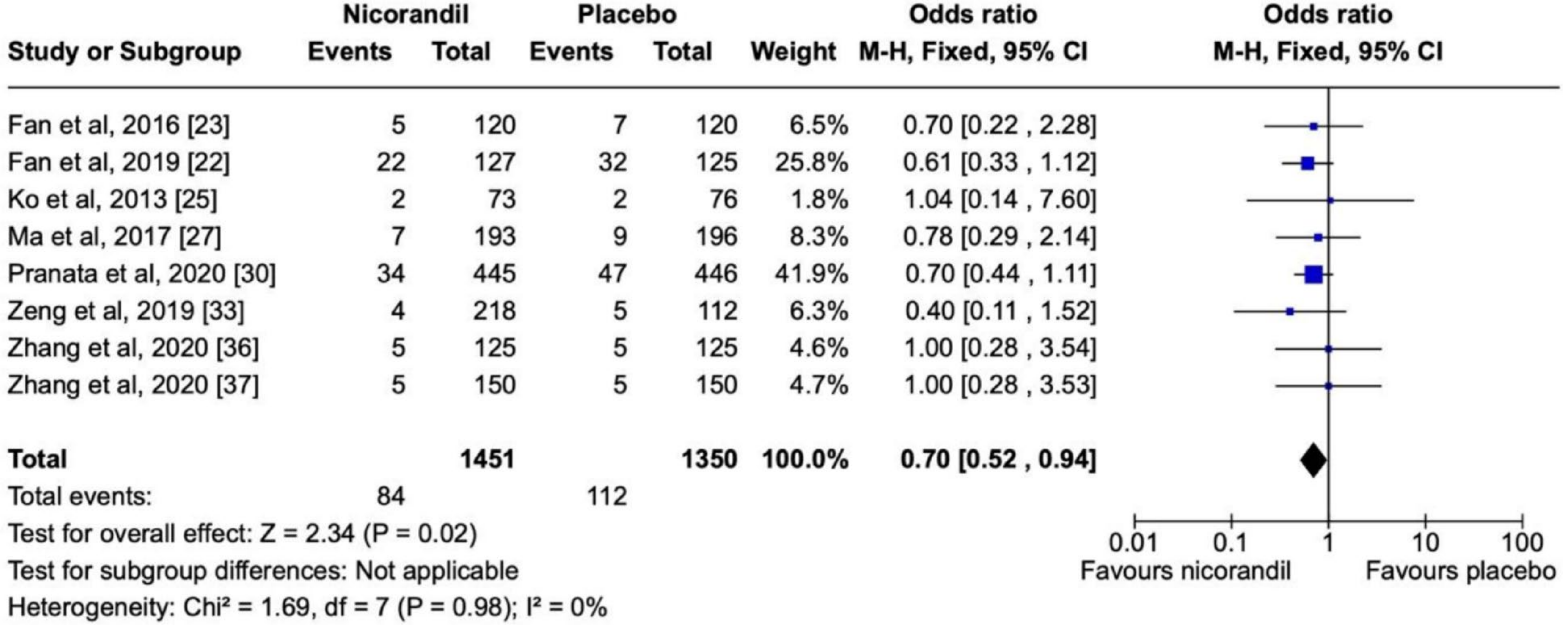
Forest plot showing statistically non-significant protective effects of Nicorandil versus placebo effect against MACE

**Fig. 7:**
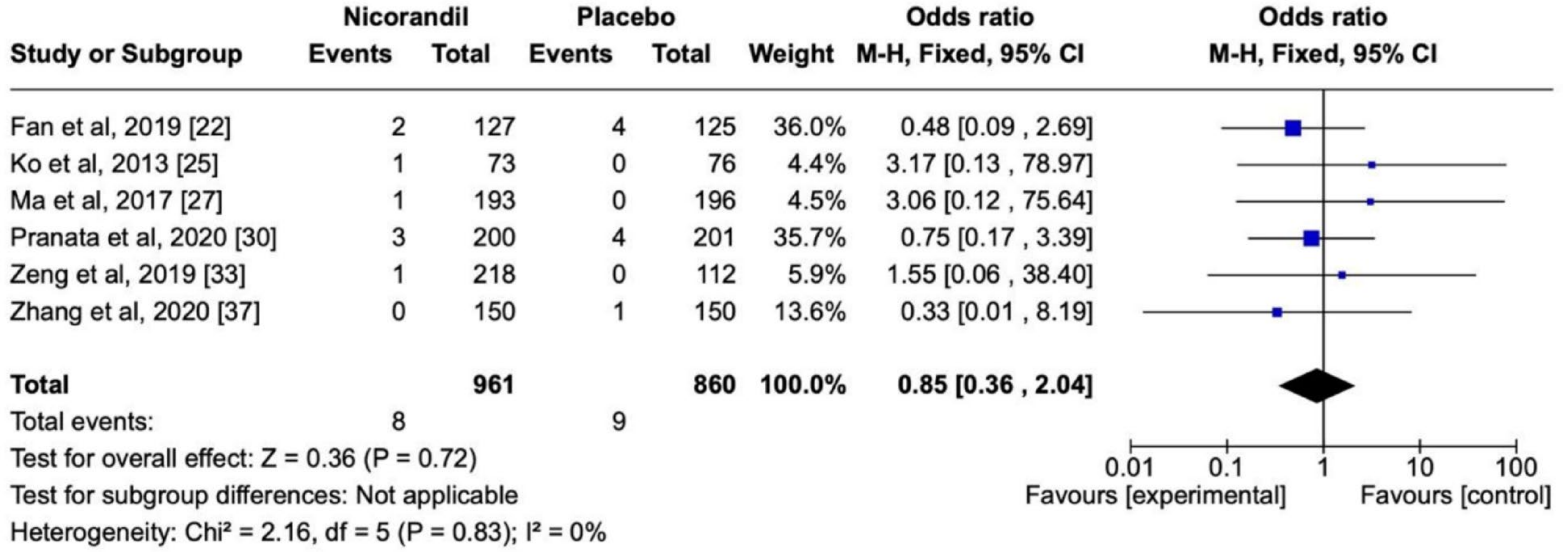
Forest plot showing non-significant protective effect of Nicorandil versus placebo against MAKE

Selected studies are shown in Table 3 and their respective forest and funnel plots in the following sequence, as per the assessed primary and secondary endpoints. Overall, the analysis showed no significant heterogeneity among the studies included. Concerning the prevention of CIN, nicorandil showed a significant protective effect in comparison with the control in terms of the overall effect (*P* < 0.00001) (Fig. 4) in a dose-dependent manner (*P* = 0.02) (Fig. 5). There was also a dose-dependent pattern in the occurrence of Major Adverse Events (MAE) (*P* = 0.02), as indicated by the Chi-Square test (Fig. 6). However, concerning the prevention of Major Adverse Kidney Events (MAKE), no robust findings contradicted the null hypothesis (*P* = 0.72) (Fig. 7). Thus, there was no significant association between MAKE and the use of nicorandil in this study.

**Table 3:** Patient demographic and characteristics data

Author	Type of Research	Population	Nicorandil group	Control group	Follow-up period after procedure	Occurrence of CIN events	Occurrence of MAE	Occurrence of MAKE
Fan et al., [22]	Prospective, open- labelled, RCT	CKD patients undergoing elective coronary procedure	*n* = 127	*n* = 125	3d-1y	8 events out of 127 in the nicorandil group	22 out of 127 in the nicorandil group	2 among 127 patients in the nicorandil group
						19 out of 125 compared to control	32 out of 125 in the control group	4 among 125 in the control group
Fan et al., [23]	Double blind, parallel RCT	Patients with impaired renal function undergoing elective PCI	*n* = 120	*n* = 120	3d-1 m	8 events out of 120 patients in the nicorandil group	5 events out of 120 patients in the nicorandil group	N/A
						21 events out of 120 patients in the control group	7 events out of 120 patients in the control group	
Iranirad et al., [24]	Prospective, open-label, RCT	High-risk patients for CIN undergoing cardiac catheterisation	*n* = 64 (10 mg nicorandil)	*n* = 64 (saline hydration)	3d	4 events out of 64 patients in the nicorandil group	N/A	N/A
						14 events out of 64 patients in the control group		
Ko et al., [25]	Investigator- initiated, prospective, open-label, multicenter RCT	Patients with renal dysfunction undergoing CAG	*n* = 73	*n* = 76	1d-2d	5 events out of 73 patients in the nicorandil group	2 events out of 73 patients in the nicorandil group	1 event among 73 patients in the nicorandil group
						6 76 patients in the control group	2 events out of 76 patients in the control group	No MAKE event among 76 patients in the control group
Li et al., [26]	MA of RCTs	Nicorandil effect on CIN prevention in patients undergoing coronary catheterisation	*n* = 363	*n* = 367	2d-3d	18 events out of 363 patients in the nicorandil group	N/A	N/A
						50 events out 367 patients in the control group		1 event out 193 patients in the nicorandil group
Ma et al., [27]	MA of RCTs	Patients who underwent eligible CAG or PCI with increased risk for CIN	*n* = 355	*n* = 354	2d	18 events out of 355 patients in the nicorandil group	7 events out of 193 patients in the nicorandil group	
						50 events out of 354 patients in the control group	9 events out of 196 patients in the control group	No MAKE event among 196 in the control group
Moghaddam et al., [28]	Open-labelled RCT	Patients at high- risk for CIN who underwent PCI	*n* = 172	*n* = 172	2d	12 events out of 172 patients in the nicorandil group	N/A	N/A
						34 events out of 172 patients in the control group		
Nawa et al., [29]	Single- center, RCT	Patients with impaired renal function who underwent PCI	*n* = 106	*n* = 107	1d	2 events out of 106 patients in the nicorandil group	*N*/A	*N*/A
						10 events out of 107 patients in the control group		
Pranata et al., [30]	SM/MA of RCTs	Nicorandil effect on CIN prevention in patients undergoing coronary catheterisation	*n* = 757	*n* = 754	2d-1y	33 events out of 757 patients in the nicorandil group	34 out of 445 patients in the nicorandil group	3 events out of 200 patients on the nicorandil group
						97 events out of 754 patients in the control group	47 events out of 446 patients in the control group	4 events among 201 patients in the control group
Sharp et al., [31]	SM/MA of RCTs	Pharmacological CIN prevention in patients undergoing cardiac catheterisation	*n* = 193	*n* = 196	Not mentioned	13 events out of 193 patients in the nicorandil group	N/A	N/A
						16 out of 196 patients in the control group		
Wang et al., [32]	MA of RCTs	Nicorandil nephroprotection against CIN	*n* = 355	*n* = 354	1d-3d	18 events out of 355 patients in the nicorandil group	*N*/A	*N*/A
						50 events out of 354 patients in the control group		
Zeng et al., [33]	RCT	Patients who underwent cardiac catheterization	*n* = 111 (DD nicorandil: 30 mg/day)	*n* = 112 (hydration)	2d-14d	6 events out of 111 patients in the DD group	2 events out of 111 patients in the DD group	No MAKE event in the DD group
			*n* = 107 (SD nicorandil: 15 mg/day)			11 events out of 107 patients in the SD group	2 events out of 107 patients in the SD group	1 event among 107 patients in the SD group
						16 events out of 112 patients in the control group	5 events out of 112 patients in the control group	No MAKE event in the control group
Zeng et al., [34]	Single- center, prospective, randomized, single-	CAD patients undergoing elective coronary procedure	*n* = 170 (DD nicorandil)	*n* = 175 (SD nicorandil)	3d	4 events out of 170 patients in the nicorandil group	N/A	N/A
	blinded study					13 events out of 175 patients in the control group		
Zhan et al., [35]	MA of RCTs	Comparison between Nicorandil, ranolazoine and control	*n* = 399	*n* = 406	1d-3d	19 events out of 399 patients in the nicorandil group	*N*/A	*N*/A
						52 events out of 406 patients in the control group		
Zhang et al., [36]	RCT	Patients with moderate renal insufficiency undergoing PCI	*n* = 125	*n* = 125	3d	2 events out of 125 patients in the nicorandil group	5 events out of 125 patients in the nicorandil group	N/A
						12 events out of 125 patients in the control group	6 events out of 125 patients in the control group	
Zhang et al., [37]	RCT	CAD patients who underwent PCI	*n* = 150	*n* = 150	2d-14d	5 events out of 150 patients in the nicorandil group	5 events out of 150 patients in the nicorandil group	No MAKE occurrence in the nicorandil group
						16 event out of 150 patients in control group	5 events out of 150 patients in control group	1 event out of 150 patients in control group
Zhao, Li and Gao, [38]	RCT	T2DM patients who underwent CAG	(*n* = 49) SD Nicorandil: 5 mg	*n* = 51	3d	3 events out of 50 patients in the DD group	N/A	N/A
			(*n* = 50) DD nicorandil: 10 mg			*4 out of 49 patients* in the SD group		
						*6 events out of 51* patients in the control group		

Selected studies concerning type of research, assessed population, intervention and control administrations, follow-up period, the occurrence of CIN, MAE and MAKE

*CIN* contrast-induced nephropathy,* MAE* major adverse event,* MAKE* major adverse kidney event,* CAD* coronary artery disease,* T2DM* type 2 diabetes mellitus,* DD* double dose,* SD* standard dose,* MA* meta-analysis,* RCT* randomised controlled trial,* PCI* percutaneous coronary intervention,* CAG* coronary angiography

### Effect of nicorandil on CIN

For this assessment, a total of 7,204 patients out of 16 papers with low heterogeneity (< 30%) (*P* < 0.00001; I2 = 0%) evaluating the effect of nicorandil on CIN showed a meaningful statistical difference in favor of the intervention group, as the occurrence of CIN was 5,14% in the nicorandil group, in contrast with 13.15% in the control group. Thus, the respective forest plot also confirmed the superior results of the intervention drug regarding CIN (Fig. 4).

### Nicorandil dose-dependent effect on CIN

The forest plot also confirmed the dose-dependent effect of nicorandil on CIN. Among 662 patients enrolled in three research types, 3,9% in the DD group presented with CIN, compared with 8,4% in the SD group. Despite the small sample size, the CI and low heterogeneity variables showed statistical reliability (*P* = 0.02; I2 = 0%) (Fig. 5).

### Nicorandil effect on MACE

This meta-analysis showed that CIN was less in patients receiving nicorandil compared to placebo group and the incidence of MACE was 5.7% and 8.2% in the Nicorandil group and the control group respectively. This difference was however statistically non-significant (*P* = 0.02; I2 = 0%). Furthermore, three studies by Ko et al. 2013, Zhang et al. 202, Zhang et al. 2019, did not show any evidence of nicorandil providing higher protection versus placebo against MACE [25, 36, 37]. The odds ratio for the use of nicorandil versus placebo using fixed effect model was 0.70 (CI 0.52–0.94).

It is also noteworthy to mention that, although authors from study [33] assessed a dose-dependent effect of nicorandil on MACE, and there was no difference between the groups. The number of MACE in the nicorandil versus SD group was 2 out of 111 and 2 out of 107 respectively.

### Nicorandil consequences in terms of MAKE

**Fig. 8:**
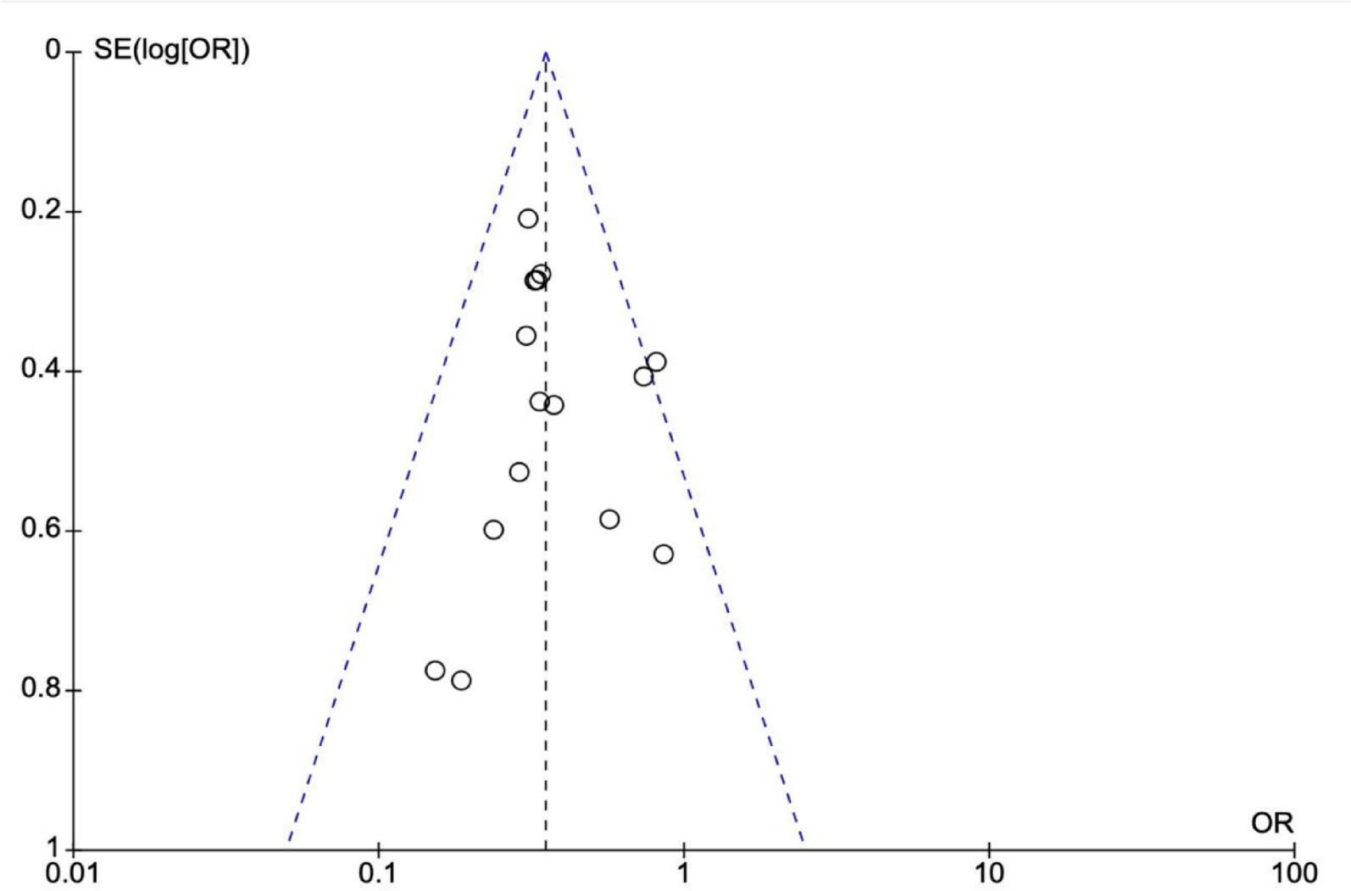
Funnel plot demonstrating no publication bias for nicorandil versus placebo against CIN, MACE and MAKE

The cross-sectional presentation of the diamond in Fig. 8 indicates that nicorandil administration could not contradict the null hypothesis. In other words, this study could not state that episodes of MAKE would be reduced by nicorandil use (Fig. 7).

**Fig. 9:**
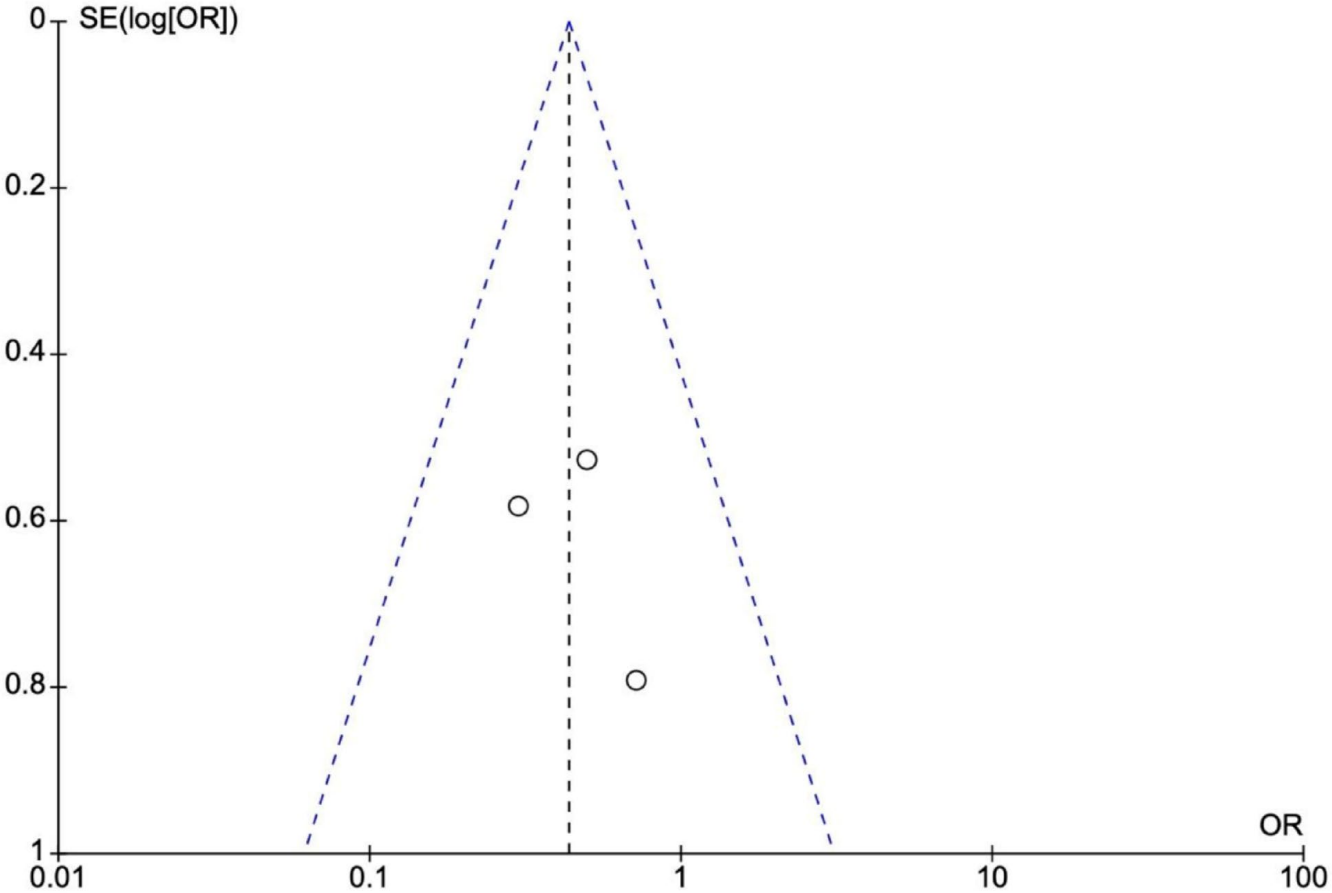
Funnel plot showing small renoprotective dose dependent effect of nicorandil versus placebo against CIN

No apparent heterogeneity was observed in these studies, and the funnel plots (Figs. 8 and 9) did not indicate publication bias. Additionally, we performed sensitivity analysis by excluding one study at a time and this did not show any significant difference in the results confirming the robustness of data and analysis.

## Discussion

This systematic review and meta-analysis, whose primary endpoint was to evaluate the efficacy of nicorandil as a nephroprotective agent against CIN in patients undergoing CAG or PCI, and the secondary endpoint was to identify the major adverse events (MAE) and major adverse kidney events (MAKE) associated with CAG or PCI after the use of nicorandil, revealed many interesting results.

In renal blood flow, only 10% of the arterial blood reaches the renal medulla, making the area more susceptible to ischaemic injury than the renal cortex upon exposure to contrast media [15]. Although the exact pathophysiological mechanism of CIN has not been fully elucidated, likely causes include contrast media-induced reduction in medullary blood flow, renal tubular cell apoptosis, and the generation of oxygen-free radicals [14, 15].

Considering that endothelial nitric oxide synthase inhibition, inflammation, and oxidative stress have all been shown to alter nitric oxide (NO) bioavailability by over 82%, it is reasonable to assume that any situation that causes an imbalance between NO and free radicals, as seen after the use of some contrast media (CM), may worsen renal cell damage [40]. Thus, antioxidants that scavenge reactive oxygen species (ROS) induced by exposure to contrast media and increase NO levels in the renal microcirculation play a critical role in preventing medullary hypoxaemia and volume depletion, and in lowering interstitial pressure [40]. Kidneys are particularly vulnerable to ischemic injury due to high metabolic and osmotic stress that can affect the intricate microvascular circulation susceptible to local and systemic hypoperfusion. This hypoperfusion or ischemia is most evident in the outer medullary region of the kidneys where the oxygen requirement is quite high. This renal ischemia risk is even higher in patients with CKD due to increased metabolic demands placed on the reduced nephron bed that also faced reduced microvascular and macrovascular circulation. It is believed that the likely pathogenesis for CIN included ischemia in the outer medullary region of kidneys exacerbated by a prolonged period of renal vasoconstriction that occurs due to imbalance of local vasoactive mediators such as adenosine, nitrous oxide, prostaglandin and reactive oxygen species, released by the vascular endothelium in response to contrast media toxicity. The resulting ischemia in tissues releasees further noxious mediators that leads to prolonged duration of vasoconstriction. Additionally, the increased viscosity of the admixture of CM and bloods plasma along with the hyperosmolality of the CM in the plasma results in further reduced blood flow, red cell distortion and aggregation contributing to significant hypoperfusion due to capillary obstruction. The combination of vasoconstriction, cytotoxicity and increased viscosity together causes significant medullary ischemic injury.

Nicorandil exerts its anti-anginal effect via two primary mechanisms: the NO pathway and the potassium channel-opening pathway [21]. However, in the context of CIN, the formal mechanism appears to be a more rational justification for its use as a CIN prophylaxis/treatment agent, as the drug itself contains a nitrate moiety that, upon reaction with the sulfhydryl group, facilitates NO release [21].

### Effect of nicorandil on contrast-induced nephropathy (CIN)

All included studies in this systematic review and meta-analysis demonstrated a significant nephroprotective effect of Nicorandil against CIN compared with controls, and the meta-analysis showed an overall nephroprotective impact of Nicorandil against CIN. The pooled OR on the effect of nicorandil in preventing CIN showed a strong statistically significant effect (Z = 11.17, p < 0.00001), where the odds of CIN were reduced by 64% with nicorandil use, implying it as a protective factor against CIN (OR – 0.36[0.30, 0.43]). Thus, oral nicorandil may be a promising adjunct to the standard hydration regimen for the at-risk population to prevent CIN. Results from similar studies supported this finding.

According to a recent study, the rate of arterial renal perfusion can independently predict the Congestive heart failure (CHF) outcomes in patients; thus, to balance the axis between the ‘total vascular organs’ (heart and kidney), the administration of intravenous infusion of Nicorandil was able to reduce the incidence of CIN [29]. However, the study noted that achieving a positive effect requires maintaining a stable nicorandil concentration in the blood during contrast exposure, as the latter has a longer half-life [29]. However, the study was limited to patients with early-stage kidney disease [29].

### Route of administration and dosage of nicorandil in renoprotection

Most of the included studies in this systematic review and meta-analysis administered nicorandil orally at 10 mg TID. However, the best route of administration (IV vs. oral) for nicorandil has yet to be determined, and more studies are needed to reach a definitive conclusion [30]. A meta-analysis showed a favourable effect of oral Nicorandil on CIN prevention in Asian patients; however, the study included only 4 RCTs (2 per arm), had wider CIs for the IV Nicorandil, and was only of moderate design quality, limiting its impact [27]. One likely explanation for this higher renal protection offered by oral nicorandil is due to its prolonged effect or bioavailability resulting in longer vasodilatation and nephroprotection. The ‘PRINCIPLE study’ by Ko et al. inferred that intravenous administration of nicorandil did not significantly decrease the incidence of CIN [25].

Furthermore, this meta-analysis demonstrated that the nephroprotective effect of Nicorandil against CIN was dose-dependent (*P* = 0.02). The forest plot comparing single-dose (SD) vs. double-dose (DD) nicorandil in our study showed a statistically significant benefit for the latter, with a 55% reduction in the odds of CIN. This finding is supported by the results of a recent study, which observed that the periprocedural use of double dose (DD) nicorandil (30 mg) significantly reduced the incidence of CIN, rather than a single dose (SD), where only a slight improvement in Renal Function Test (RFT) was seen with SD [33]. In a subgroup analysis of the use of DD IV nicorandil in the referenced study, patients who received ≥ 140 ml of contrast dye showed a significant decrease in CIN incidence compared with the standard contrast dose [33]. Thus, until more studies are available, DD Nicorandil may be beneficial for renoprotection against CIN.

### Major adverse events (MAE) and major adverse kidney events (MAKE) and the use of nicorandil

This study found that, in most studies, the occurrence of Major Adverse Events (MAE) after nicorandil use was dose-dependent and the overall result was statistically significant. Some of the MAEs that were observed with the use of nicorandil in the included studies, especially at high doses, were nonfatal MI, revascularisation, stroke, coronary artery bypass graft surgery, congestive heart failure, worsening of heart failure, pulmonary oedema, ventricular fibrillation, cardiac death, and gastrointestinal bleeding. However, this meta-analysis did not demonstrate a significant increase in Major Adverse Kidney Events (MAKE) with nicorandil use across the included studies. However, this result may be due to most of the included studies not including MAKE as an outcome. In addition, there is generally little data available on the effects of Nicorandil on MAKE. A few other studies have associated MAE, such as oral and gastrointestinal bleeding [41] and stroke [42], with the use of nicorandil. Thus, a cautious use and monitoring protocols for nicorandil should be initiated at any time for renoprotection against CIN or any other purpose.

### Limitations

None of the included studies reported the stages of chronic kidney disease (CKD) of the included patients, as this would have provided further insight into which patient category benefited most from the intervention. However, they included patients with e-GFR ˂60 ml/min. The volume and type of contrast dye used significantly affect the incidence of CIN, but most included studies failed to account for this by not providing this information. Also, the possible long-term MAE and MAKE in patients were not investigated in terms of hospital stay length, and the cost implications of using Nicorandil were not mentioned. All this information could affect the study’s outcomes.

## Conclusion

A healthy kidney is much less susceptible to potential iatrogenic complications associated with contrast media use. However, the degree of contrast media-induced acute kidney injury (AKI) and the risk of CIN vary drastically based on the presence of different harmful factors, such as advanced age, heart failure, dehydration, hypoalbuminaemia, diabetes mellitus, and anaemia. This study demonstrated that the risk of CIN in patients undergoing CAG or PCI can be reduced if nicorandil is used as a prophylaxis in addition to the standard CIN prevention protocol. Nicorandil is an effective oral medication, and its renoprotective effects are enhanced if used as a double dose. In addition, the renoprotective effect of nicorandil in this study was dose-dependent, with higher doses associated with greater MAE. Thus, while available studies have demonstrated the renoprotective effects of nicorandil in patients undergoing CAG or PCI, necessary measures and protocols should be implemented to prevent potential MAE.

Only a few studies are available on the effects of nicorandil on CIN prevention. To further generalise the potential renoprotective effects of nicorandil in patients undergoing CAG or PCI, additional studies, particularly RCTs and systematic reviews/meta-analyses, are needed. There is also insufficient information on the optimal dosage and route of administration of nicorandil in the prevention and treatment of CIN. In addition, there are limited studies on the potential renoprotective effects of nicorandil in other control conditions, such as type 2 diabetes. In fact, during the literature search, only one study reported the beneficial effect of oral Nicorandil in patients with Type 2 diabetes mellitus. Therefore, further studies are required to determine the optimal dosage, the most effective route of administration, and the effects of Nicorandil in individuals with other comorbidities.

## Data Availability

No datasets were generated or analysed during the current study.

## Abbreviations

CIN: Contrast-induced nephropathy
CAD: Coronary artery disease
CABG: Coronary artery pass graft
MI: Myocardial infarction
STEMI: ST-elevation myocardial infarction
GI: Gastrointestinal
CKD: Chronic kidney disease
PCI: Percutaneous coronary intervention
IHD: Ischemic heart disease
DALYs: Disability-Adjusted Life Years
CT-MPI: Computed tomography myocardial perfusion imaging
CCTA: Coronary CT angiography
ARI: Acute renal injury
RCM: Radiocontrast media
CM: Contrast media
MAE: Major adverse events
MAKE: Major adverse kidney events
PRISMA: Preferred Reporting Items for Systematic Reviews and Meta-Analyses
CAG: Population, intervention, comparison, and outcome (PICO) framework, coronary angiography
RCTs: Randomised controlled trials
DD: Double dose
SD: Standard dose
RoB: Risk of Bias 2
AMSTAR 2: A Measurement Tool to Assess systematic Reviews, version 2
MACE: Major adverse cardiovascular event
NO: Nitric oxide
CHF: Congestive heart failure
RFT: Renal Function Test
AKI: Acute kidney injury
